# Case report of secondary angle closure glaucoma from a Soemmering's ring presenting with episodes of transient total monocular vision loss^☆^

**DOI:** 10.1016/j.ajoc.2025.102481

**Published:** 2025-11-18

**Authors:** Sarah Zhou, Anna Urrea, Samuel J. Spiegel, Andrew K. Smith

**Affiliations:** a University of California, Irvine School of Medicine, Irvine, CA, 92697, USA; b Gavin Herbert Eye Institute, Department of Ophthalmology, UC, Irvine, California, 92697, USA; c Chicago Medical School, Rosalind Franklin University of Medicine and Science, Illinois, 60064, USA

## Abstract

**Purpose:**

To describe an interesting case of a Soemmering's ring causing intermittent secondary angle closure glaucoma presenting as painless transient monocular vision loss (TMVL).

**Observation:**

A 72-year-old male with a history of uncomplicated cataract surgery presented with episodes of TMVL in the right eye, lasting minutes, and without other associated symptoms. His initial intraocular pressure (IOP) was normal, and after neuro-ophthalmology assessment he underwent work-up for amaurosis fugax which was unrevealing. At follow up, the patient was found to have an IOP of 40 mmHg. Gonioscopic examination showed no visible angle structures and ultrasound biomicroscopy revealed a large Soemmering's ring and no pseudophacodonesis. Laser peripheral iridotomy (LPI) was performed resulting in return of IOP to the patient's baseline along with resolution of TMVL episodes.

**Conclusion and importance:**

We describe a rare presentation of painless recurrent TMVL resulting from secondary angle closure caused by a Soemmering's ring. This case highlights the importance of retaining intermittent IOP elevation on the differential diagnosis for TMVL.

## 1 Introduction

Intermittent angle closure often presents with signs of acute angle closure crisis associated with elevated intraocular pressure (IOP) such as eye pain, headache, blurred vision, halos around lights, nausea and vomiting.^1^ However, it can also cause episodes of amaurosis fugax.^2^ Amaurosis fugax is defined as transient monocular vision loss (TMVL) that typically lasts minutes due to ischemia or vascular insufficiency and generally has full recovery.^1^ In some cases, there may be no ophthalmoscopic evidence of ischemia. Evaluation of the etiology of TMVL includes assessment for sources of a transient ischemic attack (TIA), which is a self-resolving, transient neurologic deficit secondary to focal ischemia of the central nervous system or retina. Assessment should include electrocardiogram, echocardiogram, evaluation of the carotid artery via ultrasound, computed tomography angiogram, or magnetic resonance angiography (MRA) as a TIA can be a sign of an impending stroke.^1^^,^^3^^,^^4^ Amaurosis fugax is considered equivalent to a TIA as it results from focal ischemia in a vascular distribution which is proximally shared by the CNS.^1^ Common etiologies of amaurosis fugax include ipsilateral carotid embolism, cardioembolism, or a thrombus due to a hypercoagulable state.^3^ However, TMVL can also be caused by a variety of conditions such as giant cell arteritis, vasospasm, papilledema, ocular ischemic syndrome, and intermittent elevation in intraocular pressure (IOP).^1^^,^^5^^,^^6^

Intermittent IOP elevation can occur when the iridocorneal angle is temporarily obstructed by lens-iris apposition in phakic patients causing pupillary block; however, angle closure can also rarely occur in pseudophakic patients.^7^ Pseudophakic angle closure may be secondary to ciliary body or iris mass, medication-induced ciliochoroidal effusions, aqueous misdirection, neovascular glaucoma, or intraocular lens-iris adhesions among others. Recognizing the underlying cause of secondary angle closure is key to reestablishing aqueous outflow through the pressure-dependent pathway.

One cause of secondary angle closure previously reported in pseudophakic and aphakic patients is a Soemmering's ring.^8^, ^9^, ^10^, ^11^ A Soemmering's ring occurs after cataract surgery when retained epithelial lens cells proliferate along the periphery of the capsule to form a ring of cortical fibers.^12^ Soemmering's rings are usually asymptomatic; however, cases of secondary angle closure glaucoma, corneal decompensation, uveitis-glaucoma-hyphema (UGH) syndrome, and growth into the visual axis have been reported.^8^, ^9^, ^10^^,^^13^, ^14^, ^15^

In this case report, we describe a patient who presented with painless TMVL episodes as his only symptom and was found to have intermittent angle closure glaucoma secondary to a Soemmering's ring which was relieved by laser peripheral iridotomy (LPI).

## 2 Case report

**Fig. 1:**
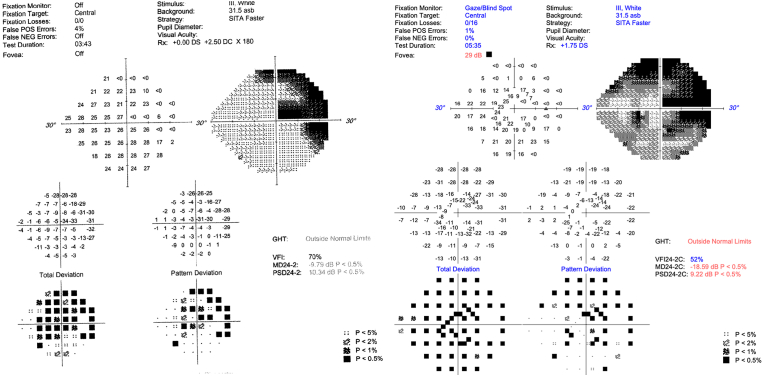
Visual field of the right eye two years prior to presentation demonstrating a superior arcuate defect and early inferior arcuate defect (A). Visual field of the right eye at presentation demonstrating progression of superior and inferior arcuate defects (B). Of note, testing in (A) utilized the Swedish Interactive Thresholding Algoritim (SITA) Faster 24-2 test while testing in (B) utilized the SITA 24-2C test.

A 72-year-old Caucasian male presented with painless, transient “blacking out” of vision in his right eye. He had no past medical history of smoking, obesity, hypertension, hyperlipidemia, diabetes mellitus, prior thrombus, or obstructive sleep apnea. The patient had a history of phacoemulsification with intraocular lens implantation twelve years prior to presentation without reported surgical complications. Prior to symptom onset, the patient had been followed by the retina service for advanced nonexudative age-related macular degeneration (AMD) and pigmentary retinal dystrophy. Visual field testing two years prior to presentation showed an enlarged blind spot and temporal visual field loss (Fig. 1). Full-field electroretinogram in at that time showed severe photoreceptor dysfunction in both eyes.

At a follow-up retina visit, the patient described three episodes of total blacking out of the vision in the right eye in a single day, each lasting less than 2 min and spontaneously resolving. Stroke workup was initiated, including magnetic resonance imaging of the brain and orbit as well as MRA of the head and neck. No acute findings nor stenosis were demonstrated. An echocardiogram also did not show potential sources of emboli. Erythrocyte sedimentation rate and C-reactive protein values were within normal limits. After negative workup, in the setting of the patient's known pigmentary retinal dystrophy with visual field loss, his symptoms were presumed to be from difficulty adapting to changes in ambient lighting.

However, a few months later, the patient began having daily episodes of vision dimming in his right eye lasting on the order of seconds. He reported that his vision would darken to the point of only being able to make out shapes. At a follow-up neuro-ophthalmology visit, the patient was incidentally found to have an IOP of 40 mmHg in the right eye. He was not experiencing blurry vision, headache, nausea or vomiting at the time. The patient was started on latanoprost and a dorzolamide-timolol combination drop and was promptly referred to the glaucoma service.

At presentation to the glaucoma service, best corrected visual acuity was 20/40 in both eyes, and the patient denied current symptoms of headache, nausea, vomiting, or halos around lights. Pupils were both round and reactive to light. Intraocular pressure was 45 mmHg in the right eye on latanoprost and dorzolamide-timolol and 11 mmHg in the left eye. The right eye anterior chamber was noted to be slightly shallower than the left with peripheral elevation of the iris. No pseudoexfoliative material nor pseudophacodonesis were observed. The optic nerves were myopic-appearing with temporal tilting and peripapillary atrophy (PPA), and the cup-to-disc ratio of the right and left eyes were 0.8 and 0.4, respectively. On gonioscopic evaluation, the angle of the right eye was open only to Schwalbe's line (Grade I) in all quadrants while the left eye was open to ciliary body (Grade IV) in all quadrants.

**Fig. 2:**
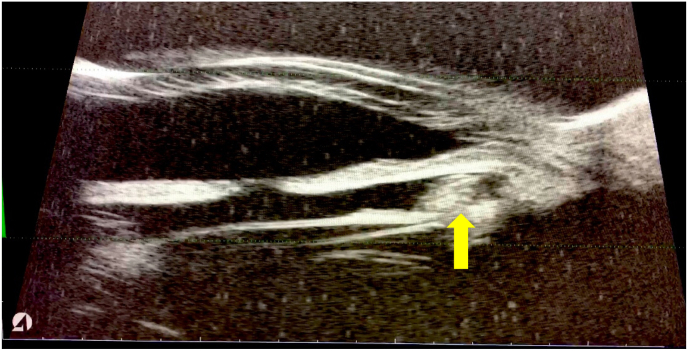
Ultrasound biomicroscopy of the right eye demonstrating a hyperechoic mass (yellow arrow) behind the iris causing anterior displacement of the iris and iridocorneal angle occlusion. A single cut is shown, however the mass was circumferential on UBM exam. (For interpretation of the references to colour in this figure legend, the reader is referred to the Web version of this article.)

Optical coherence tomography (OCT) of the optic nerve showed poor segmentation and diffuse nerve fiber layer thinning in both eyes and repeat visual field analysis revealed progression of visual field loss in the right eye (Fig. 1). The anterior chamber depth (ACD) was measured using the Zeiss IOLMaster® 700 (Carl Zeiss Meditec AG, Jena, Germany) and found to be 3.35 mm in the right eye and 4.38 mm in the left eye. B scan ultrasonography showed no masses in the posterior segment. Ultrasound Biomicroscopy (UBM) of the right eye showed a circumferential cystic mass emanating from the intraocular lens causing elevation of the peripheral iris and narrowing of the iridocorneal angle (Fig. 2). The patient's pupil was poorly dilated to 4 mm, preventing direct and indirect visualization of the ciliary sulcus mass with a gonioscopic lens on slit lamp exam.

The patient was administered topical brimonidine and netarsudil and was given 500 mg of oral acetazolamide, and the IOP decreased to 25 mmHg. The patient returned the next day for LPI. His pre-procedure IOP was 35 mmHg, which decreased to 15 mmHg after two LPIs were performed 180° apart.

**Fig. 3:**
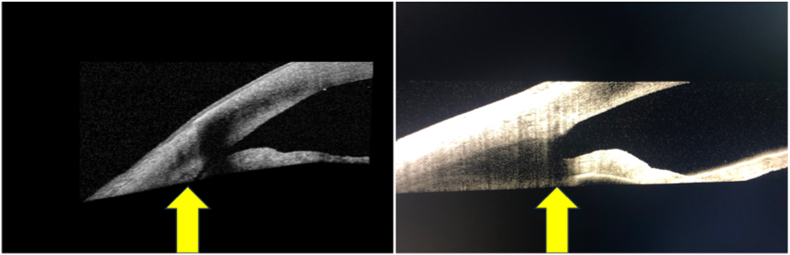
Anterior segment optical coherence tomography (AS-OCT) of the right eye before (left) and after (right) LPI showing widening of the iridocorneal angles (yellow arrows). Anterior chamber depth was 3.38 mm before and 4.38 mm after the procedure. (For interpretation of the references to colour in this figure legend, the reader is referred to the Web version of this article.)

Repeat examination post-LPI showed a deepened anterior chamber that was now similar to the left eye, and gonioscopy showed the angle of the right eye open to ciliary body (grade IV) with 3 small areas of peripheral anterior synechiae. Repeat measurement of ACD with the IOLMaster® 700 showed an increase from 3.35 mm to 4.38 mm. Anterior segment OCT also showed an open iridocorneal angle and a more posterior iris (Fig. 3).

After the procedure, the patient was continued on latanoprost and dorzolamide-timolol drops in the right eye and was seen regularly throughout the following year. At 12 months' follow-up, the patient's IOP consistently measured in the normal range, gonioscopic exam remained stable, and the patient continued to endorse relief from prior episodes of TMVL.

## 3 Discussion

Soemmering's rings are a type of secondary cataract and are part of the spectrum of posterior capsular opacification (PCO) that develops from proliferation of residual lens epithelial cells after cataract surgery.^11^^,^^12^^,^^16^ They are classically a donut-shaped material located at the equator of the capsular bag and have been shown to develop within the first 2–5 years postoperatively, but complications from Soemmering's rings can manifest much later.^8^^,^^11^^,^^13^ Patient-dependent risk factors for developing a Soemmering's ring include early (childhood) lensectomy, diabetes, and pre-existing ocular disease including dry eye and uveitis.^17^ Other risk factors for developing a Soemmering's ring include capsule removal during cataract surgery, lens material, and lens shape.^17^ Complications from Soemmering's rings include dislocation of the Soemmering's ring, UGH syndrome, corneal edema, and angle closure in aphakic and pseudophakic eyes.^8^^,^^10^^,^^13^, ^14^, ^15^^,^^18^, ^19^, ^20^

We present a case of pupillary block secondary angle closure due to a Soemmering's ring presenting as TMVL that manifested 12 years after uneventful cataract surgery in a patient with a history of advanced AMD and retinal pigmentary dystrophy. TMVL is a unique presenting symptom to have for angle closure as, generally, acute angle closure attacks present with blurred vision rather than total blacking out of vision and are associated with orbital or periorbital pain. While TMVL, painless vision loss, and amaurosis fugax are sometimes used interchangeably, total blacking out of vision is usually associated with amaurosis fugax, which needs a neurologic and systemic ischemic workup for a TIA. In this case, we see that the symptom of amaurosis fugax was instead associated with intermittent angle closure and resulting elevated IOP—pathologies that can also decrease retinal perfusion and affect visual acuity.^2^^,^^21^ The patient underwent a thorough neuro-ophthalmologic workup for a TIA with negative imaging and laboratory tests. Confounding factors on the patient's exam that initially were not indicative of intermittent angle closure were lack of pseudophacodonesis and active pigmentary cell, no documented episodes of IOP elevation, myopic-appearing nerves with peripapillary atrophy that made it difficult to assess optic nerve cupping, and subtle differences in ACD due to more peripheral shallowing.

Transient total vision loss from elevated IOP is thought to occur from a lack of ocular perfusion. The ocular perfusion pressure (OPP) is equal to the mean arterial pressure (MAP) minus the IOP (OPP = MAP – IOP).^22^ Around the time of the patient's initial symptoms, MAP was found to be 90 mmHg; therefore, the IOP would temporarily have to exceed 90 mmHg in order to exert the TMVL the patient experienced. However, taking into consideration the retinal atrophy and photoreceptor dysfunction seen in advanced AMD and pigmentary retinal dystrophies, the patient may have experienced a more severe degree of vision loss at a lower IOP due to reduced capacity of total remaining photoreceptors to withstand fluctuations in retinal perfusion during episodes of elevated IOP.^23^^,^^24^ Although it is possible that the patient's IOP was temporarily exceeding 90 mmHg during the described “blacking-out” episodes, it is also possible that a transient IOP over 90 mmHg would not be necessary to cause TMVL in this case given the retina's dystrophic status.

Secondary angle closure after cataract extraction is rare, but the underlying cause may be serious. Case reports have described secondary angle closure due to retained lens material or viscoelastic, Soemmering's rings, and aqueous misdirection.^11^^,^^13^^,^^25^, ^26^, ^27^ Angle closure in pseudophakic patients may be caused by non-pupillary block and pupillary block mechanisms. Non-pupillary block angle closure in pseudophakia includes aqueous misdirection and mechanisms that lead to the formation of peripheral anterior synechiae (PAS) such as inflammation, prolonged shallowing of the anterior chamber, or iris incarceration.^10^^,^^15^^,^^28^ Pseudophakic pupillary block, similar to phakic pupillary block, occurs when the intraocular lens and pupil margin are brought into apposition, often precipitated by material in the capsular bag such as lens fragments, viscoelastic, or proliferative lens material.^10^^,^^26^^,^^27^ Angle closure after uneventful cataract surgery may also manifest years after surgery, and therefore, workup of a pseudophakic patient in angle closure requires careful history-taking and examination.^13^^,^^29^ Given the sometimes indolent development of secondary angle closure, it is important to rule out the interval development of an iris or ciliary body malignancy that may also present as peripheral iris elevation and angle closure.^8^ Conversely, when evaluating patients for possible iris or ciliary body malignancy, it is also important to take into account the patient's surgical history and to include post-surgical causes of peripheral iris elevation on the differential diagnosis.^8^ In addition to history and examination, imaging behind the iris is also a key diagnostic tool to characterize the cause of secondary angle closure.^10^

Important diagnostic data involved in the workup were gonioscopy and UBM, which demonstrated angle closure in the affected eye and a circumferential circular hyperechoic structure behind the iris (Fig. 2). The finding of a uniformly hyperechoic structure that was circumferentially present behind the iris was reassuring for Soemmering's ring over ciliary body mass.^8^^,^^30^ The patient's elevated IOP, angle closure, and episodes of blacking out were all improved after LPI. Two iridotomies were fashioned 180° apart in order to overcome loculations in aqueous between regions of the Soemmering's ring that prevented successful relief of pupillary block with the first iridotomy.

In cases of pupillary block angle closure induced by Soemmering's ring, the anatomic cause of pupillary block differs from that of phacomorphic pupillary block induced by a native crystalline lens. In phacomorphic angle closure, the area of lens elevation and potential lens-iris apposition occurs in the mid-periphery of the iris whereas in Soemmering's ring-induced angle closure, the area of elevation and iris apposition to the Soemmering's ring is more peripheral (Fig. 2). Therefore, the LPI in Soemmering's ring-induced angle closure should be placed more peripherally, outside the borders of the Soemmering's ring. Other case reports have described whitish material moving into the anterior chamber after LPI or needing additional laser photodisruption of the lens capsule through the iridotomy, demonstrating possible contribution from the lens capsule or more peripheral parts of the Soemmering's ring to the pupillary block.^10^^,^^11^

In previous cases where patients presented with gradual insidious vision loss and elevated IOP, the process of angle closure may have been more similar to chronic angle closure with gradual growth of the Soemmering's ring.^10^^,^^11^ In our case, the intermittent nature of the angle closure could be related to changes in iris configuration with pupil dilation in dim light settings such as would occur in phacomorphic pupillary block. It could also be related to positional changes of a mobile Soemmering's ring itself; however, the lack of pseudophacodonesis on exam would make this less likely.

In conclusion, we present a case of intermittent angle closure 12 years after uneventful cataract extraction presenting as TMVL. While TMVL is most commonly embolic in etiology, our case demonstrates the importance of considering less-common etiologies such as intermittent IOP elevation from secondary angle closure particularly in pseudophakic patients.^31^^,^^32^

## CRediT authorship contribution statement

**Sarah Zhou:** Writing – review & editing, Writing – original draft. **Anna Urrea:** Writing – review & editing. **Samuel J. Spiegel:** Writing – review & editing. **Andrew K. Smith:** Writing – review & editing, Writing – original draft, Conceptualization.

## Patient consent

Written consent to publish this case has not been obtained. This report does not contain any personal identifying information.

## Acknowledgements and disclosures

Research reported in this publication was supported by an unrestricted grant from Research to Prevent Blindness to the Gavin Herbert Eye Institute at the University of California, Irvine.

## Authorship

All authors attest that they meet the current ICMJE criteria for authorship.

## Declaration of competing interest

The authors declare that they have no known competing financial interests or personal relationships that could have appeared to influence the work reported in this paper.
